# Venous versus capillary sampling for total creatine kinase assay: Effects of a simulated football match

**DOI:** 10.1371/journal.pone.0204238

**Published:** 2018-09-20

**Authors:** Donizete C. X. de Oliveira, Ariobaldo Frisselli, Edirley G. de Souza, Luiz Cláudio R. Stanganelli, Rafael Deminice

**Affiliations:** 1 Department of Physical Education, Faculty of Physical Education and Sport, State University of Londrina, Londrina, Paraná, Brazil; 2 Department of Sport Science, Faculty of Physical Education and Sport, State University of Londrina, Londrina, Paraná, Brazil; Sao Paulo State University - UNESP, BRAZIL

## Abstract

**Background:**

Capillary rather than venipuncture may be a simpler and less invasive blood collection protocol that would increase the number of potential sampling tests. However, if capillary sampling can be used as an alternative to venipuncture to determine changes in plasma, total creatine kinase (CK) activity in response to a football training session is poorly known.

**Objective:**

This study aims to determine whether capillary blood sampling would provide representative measures of total CK activity compared to venipuncture in response to a football training session-induced elevated CK plasma levels.

**Methods:**

Twenty-two players from an under-19 football team performed a simulated football match with 11 players on each team for 90 minutes total duration (two halves of 45 minutes with 15 minutes rest between). Venous and ear lobe capillary blood samples were collected before and after (24h and 48h) the training session. Athletes retested for three consecutive days after exercise during the recovery week.

**Results:**

The simulated match significantly increased (*P*< 0.05) total CK activity as determined in both venous (1.7-fold) and capillary (1.9-fold) blood sampling. Total CK activity determined using capillary samples demonstrated significant correlation (r = 0.85; *P* < 0.01) and an elevated concordance *Lin* index (*pc* = 0.80) when compared to venous sampling total CK. The Bland–Altman plot showed capillary sampling CK overestimated venous CK levels by 130 U/L (61%), with moderated variance and low bias.

**Conclusions:**

Our results demonstrated that capillary sampling for total CK activity assay may be considered a reliable alternative to venipuncture to determine changes in plasma total CK activity in response to a football training session.

## Introduction

Capillary blood samples taken by finger prick or ear lobe are largely used to determine several compounds (e.g., glucose, potassium, phosphorous, sodium, bilirubin) in medicine [1–3]. Capillary, rather than venipuncture, would provide a simpler and less invasive blood collection protocol that requires low-cost materials and would increase the number of potential sampling tests with minimally trained individuals [3]. It is especially important regarding the sports field, since blood sampling may happen in between training sets or game matches at the football field, swimming pool or any sports location [4]. Indeed, capillary puncture allows the collection of successive blood samplings with minimal disturbance to athletes’ routines.

Because it is related to muscle damage, plasma total creatine kinase (CK) levels have been recently and extensively studied as an indirect skeletal muscle damage marker following physical effort, especially in sports [5,6]. Studies have demonstrated that intensive exercise causes a greater disruption or injury to the muscle tissues that may cause CK to leak from cells into blood serum [7,8]. As such, high increases in serum total CK have been demonstrated after match play or intense exercise in different sports modalities for up to 72 hours [9–12]. Moreover, reduced power and strength as well as elevated delayed-onset muscle soreness have been observed for up to 48 hours alongside elevated CK plasma concentration following elite football competition [10]. Indeed, total CK has been used as an indicator of player fatigue, an early indicator of an athlete’s injured skeletal muscle, and a potential monitor of football player recovery status [12,13]. However, whether capillary blood sampling would provide a simpler reliable tool to measures of total CK activity compared to venipuncture in response to a high intensity exercise such as a football training session is poorly known. We first hypothesized that capillary blood for total CK measurements could be a reliable alternative to venipuncture, which would be helpful to coaches and athletes, since capillary blood sampling is easier and could be made with slight disturbance to athletes.

Therefore, we aimed to determine the reliability of plasma total CK activity determined using capillary *vs* venous blood sampling in response to a football training session that promoted increased total CK plasma concentration.

## Methods

The volunteers participating in the present study were 22 healthy and well-trained males aged 16.7±1.0 years. All subjects were under-19 football players from the same team and were well trained contesting the second division of the Parana state championship. The protocol was approved by the Research Ethics Committee of the State University of Londrina and was according to the Helsinki Declaration. All volunteers gave written informed consent and agreed to voluntarily participate in the study. None of the participants smoked or were taking any type of medication.

All the athletes were invited to attend the Laboratory of Exercise Biochemistry of the State University of Londrina three days in the same week. On the first day (Pre), the basal levels of venous and capillary blood were collected. The participants were then submitted to a simulated football match with 11 players on each team and 90 minutes total duration (two halves of 45 minutes with 15 minutes rest between). The simulated match contained refereeing and organization by the team's own coaches. Then, venous and capillary blood samples were taken at 24 hours and 48 hours after the simulated match. The goalkeepers were excluded from the blood sampling procedures and three of the athletes did not complete all the blood sampling and were excluded from the study. A total of 17 were included in the study. All tests and collections were performed by the same researchers at the same periods of the day and rest, as well as the same feeding conditions being continued throughout the experiment.

Venous blood was collected using a 4 mL heparinized vacutainer^®^ tubes at antecubital vein puncture. An ear lobe puncture was used for capillary blood collection using heparinized capillary tubes. A total of 100 μl of blood was collected using two capillary tubes of 50 μl each that were then transferred to heparinized Eppendorf tubes. Venous and capillary blood sampling was performed at the same time for two different people. Venous and capillary blood tubes were kept refrigerated at 4 °C until the end of each trial (~30 min) and later centrifuged at 1000 g for 15 minutes at 4 °C. Plasma was stored in Eppendorf tubes at -80°C for later analysis.

Plasma total CK enzyme activity of capillary blood sampling was the same used for vein-puncture samples and followed the commercial kit instructions from Labtest (Lagoa Santa, Minas Gerais, Brazil) measured using the commercial kit from Labtest (Lagoa Santa, Minas Gerais, Brazil) in a plate reader from Epoch (BioTek instruments, Winooski, VT, USA).

The intra- and inter-assay’s coefficient of variation was less than 5% for all analyses (intra-assay for venous CK 4.3% and for capillary CK 4.9%) and (inter-assay for venous CK 4.0% and for capillary CK 4.1%).

Data are reported as mean ± SEM. A linear mixed effects model was used to detect possible differences between total CK activity determinations using venous versus capillary sampling at different sampling times (pre-exercise and 24 hours and 48 hours after exercise). Student’s t test was used to determine possible differences between total CK activity areas under curve (AUC) determined using venous versus capillary sampling. The Pearson correlation coefficient, Lin’s concordance coefficient, the Bland–Altman plot inspection and Intraclass Correlation Coefficient (ICC) were used to evaluate association, concordance, reproducibility and reliability between venous versus capillary sampling, respectively. In the Bland–Altman plot, venous sampling total CK activity was used as a reference method. The coefficient of variation (CV) was also determined to evaluate repeatability. The level of significance was set at *P*< 0.05 in all analyses.

## Results

**Fig 1:**
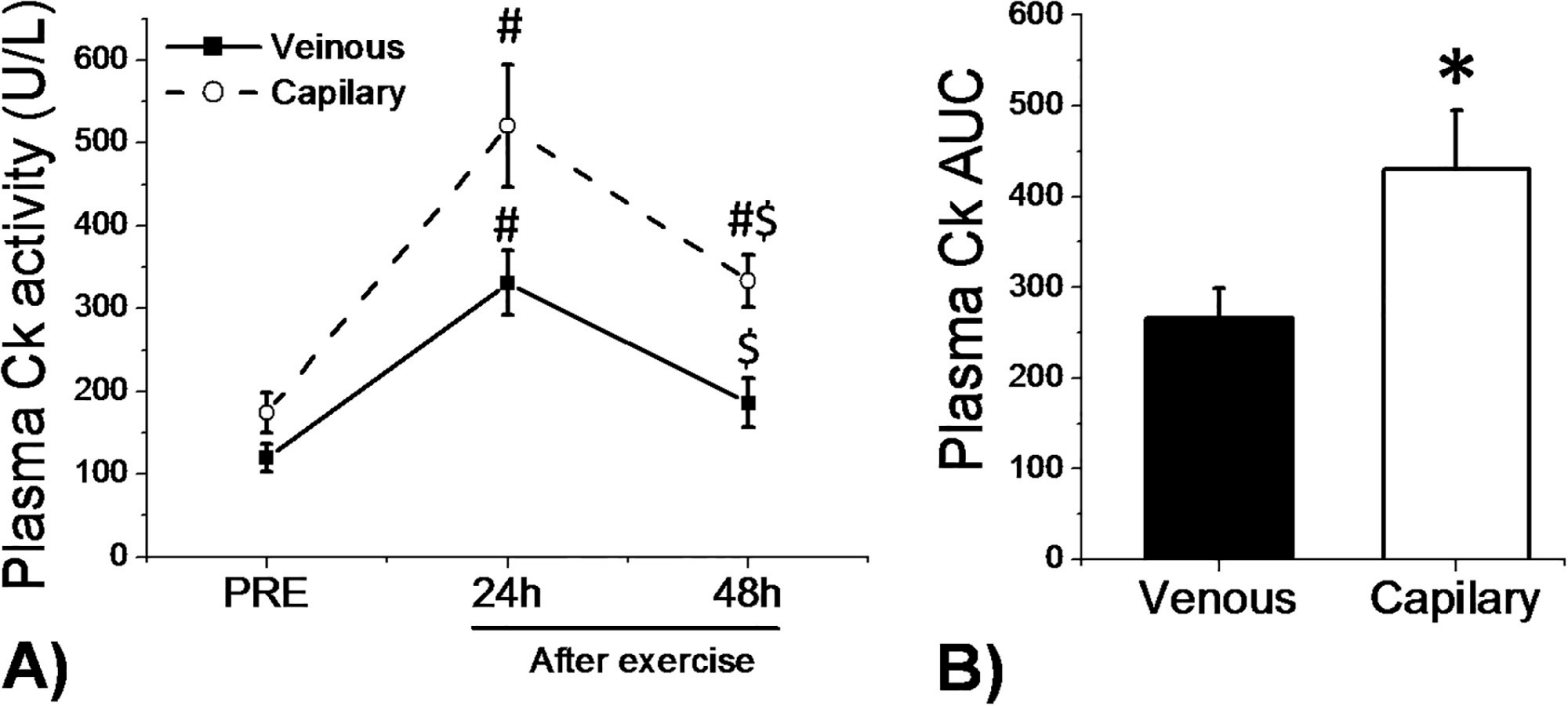
Behavior of plasma total CK determined using venous and capillary sampling pre-exercise and 24h and 48h after a training session (A) (# indicates significant difference compared to Pre; $ indicates significant difference compared to 24h; *P* < 0.05 by linear effects mixed model). Plasma total CK area under curve (AUC) determined using venous and capillary sampling (B) (* indicates significant difference compared to venous sampling; *P* < 0.05 by Student’s t test).

Fig 1 presents the total CK activity determined pre-exercise and after exercise (24 hours and 48 hours) using the two different sampling techniques, venous and capillary. The training session significantly increased (*P*< 0.05) total CK activity as determined by both venous (1.7-fold) and capillary (1.9-fold) blood sampling at 24 hours after exercise. Total CK plasma concentration returned to pre-exercise levels at 48 hours after the training session when the venous sampling of the total CK curve was analyzed. This result was different from the capillary sampling curve that remained elevated until 48 hours after the training session. These differences were evident when comparing total CK activity AUC that was significantly higher (*P*< 0.05) in capillary when compared to venous sampling (Fig 1).

**Fig 2:**
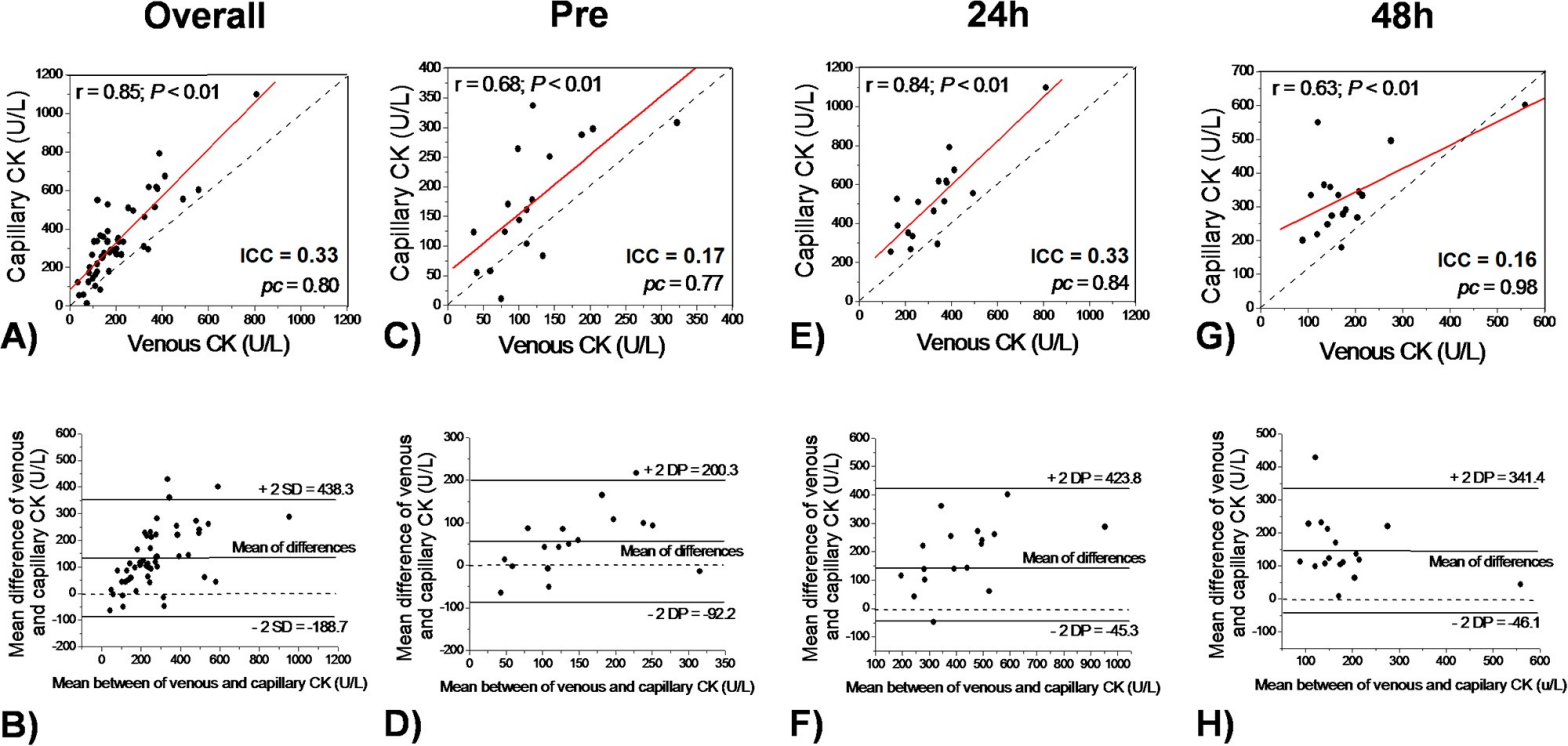
Pearson correlation coefficient (*r* and solid line), Lin’s coefficient (*pc* and dash line) and Bland–Altman plot of total CK determined using venous and capillary sampling for overall (A and B), Pre (C and D) and 24h (E and F) and 48h (G and H) after training session.

Overall, total CK activity that was determined using capillary samples demonstrated significant correlation (r = 0.85; *P* < 0.01) and an elevated concordance Lin’s index (*pc* = 0.80) when compared to venous sampled total CK. However, the Bland–Altman plot showed capillary sampled total CK overestimated venous total CK levels by 130 U/L (61%), with moderate-elevated variance and bias. A small ICC (0.33) and elevated CV (68 and 60% for venous and capillary sampling, respectively) were also demonstrated for overall comparation between venous and capillary total CK determination (Fig 2). When analyzed separately, capillary sampled total CK demonstrated significant correlation (Pre-r = 0.68, *P*< 0.01; 24hr = 0.84, *P*< 0.01; 48hr = 0.63, *P*< 0.01) and elevated concordance Lin’s index (Pre-pc = 0.77; 24h *pc* = 0.84; 48h *pc* = 0.98) for all sampling times. Moreover, capillary overestimated venous total CK in all sampling times studied, ranging from 45% to 79%. A small ICC (0.17, 0.33 and 0.16 for pre-exercise and 24 hours and 48 hours after exercise, respectively) and elevated CV (from 35 to 57%) were also demonstrated when analyzed different time sampling (Fig 2).

## Discussion

The results of our study demonstrated that capillary sampling may not be considered a reliable alternative to venipuncture to determine changes in plasma total CK activity in response to a football training session. It is important to point out that although the elevated correlation and concordance demonstrated between both sampling methods, reproducibility and reliability are low and total CK activity measured using capillary blood sampling may overestimate those determined in venous blood sampling by ~60%.

Total CK has been extensively used by football physiologists and coaches to monitor fatigue and recovery status throughout the competitive season [5,12,13]. Taking this into account, capillary blood sampling for total CK assay may present some advantages over the traditional venous sampling, especially regarding the football competitive routine. Knoblauch et al. [4] characterized capillary sampling as a simpler and less-invasive technique, which allows a higher number of samplings during the day, week or competition season without disturbing the athletes’ routine, while also being a lower-cost method. Few previous studies have demonstrated capillary as a valid alternative to venous sampling to measure total CK activity. Nunes et al. [3] first demonstrated finger puncture as a reliable method to measure several resting hematological and biochemical parameters in soccer and handball players, including total CK. These authors demonstrated a striking Pearson correlation coefficient of 0.99 between capillary and venous sampling measured total CK activity. Knoblauch et al. [4] also demonstrated an elevated correlation of total CK activity between two sampling techniques (0.99) after resistive exercise in untrained men and women college students. However, none of the studies cited above presented concordance, agreement or reliability analysis. Also, although Knoblauch et al. [4] describes having measured total CK activity prior and 0, 24, 48, 72, and 96 hours after exercise, these authors did not present the results or any correlation, concordance or agreement analysis among them. Thus, to the best of our knowledge, this is the first study to demonstrate capillary is not as a reliable alternative to venipuncture to determine changes in plasma total CK activity induced by a session of exercise training using a timeframe analysis. This decision was based on the small levels of reproducibility and reliability demonstrated between venous and capillary for all time sampling. According to Koo and Li [14], *Pearson* correlation coefficient is only a measure of correlation, and hence, they are non-ideal measures of reliability. We believe therefore that compare venous vs capillary sites with agreement and reliability measurements (Lin coefficient, Bland & Altman plot and Intraclass Correlation Coefficient) is important, novelty and brings a new interpretation to the data.

In addition to low agreement and reliability between both blood sampling techniques used for total CK assay in the present study, the elevated (~60%) overestimation of venous blood sampling by capillary sampling must be considered, especially regarding practical applications. Nunes et al. [3] attributes the differences found to the peripheral ultra-filtration of intravascular fluid, which may lead to hemoconcentration of capillary blood punctures. Therefore, it is reasonable to say that capillary sampling for total CK determination can be useful when establishing individual baseline measures or an individual profile over a significant timeframe; so, decisions regarding individual athlete responses can be made. The use of capillary blood total CK measurements to compare athletes using different blood sampling techniques must be avoided. In addition, capillary blood sampling for total CK measurements cannot be used to compare with total CK pre-established cut-points as proposed by Mougios [15] and Inman et al. [16], which may generate interpretation errors.

## Conclusion

In conclusion, capillary blood sampling cannot be used as a reliable alternative to venipuncture to evaluate changes in plasma total CK activity induced by a football training routine. The low reproducibility and reliability between methods may lead coaches and physiologists to make incorrect data interpretations. It is important to consider however the usage of capillary sampling for total CK determination when establishing individual baseline measures in future studies.

## Supporting information

S1 File (PDF)
