# Supplementary material for: Venous versus capillary sampling for total creatine kinase assay: Effects of a simulated football match
Source: PLoS One. 2018 Sep 20;13(9):e0204238. doi: 10.1371/journal.pone.0204238 (PMC6147722; doi:10.1371/journal.pone.0204238)
Supplement: S1 File — (PDF) [file pone.0204238.s001.pdf]

	Pre			
	antecubital vein	ear lobule	Chance	mean
J13	120,00	336,92	216,92	228,46
J17	100,51	143,59	43,08	122,05
J19	41,31	55,08	13,77	48,20
J2	133,89	83,37	-50,53	108,63
J21	119,08	177,71	58,63	148,40
J22	98,93	263,82	164,89	181,37
J24	36,92	123,08	86,15	80,00
J7	111,04	161,19	50,15	136,12
J10	143,28	250,75	107,46	197,01
J14	204,18	297,31	93,13	250,75
J15	75,22	10,75	-64,48	42,99
J20	85,16	170,32	85,16	127,74
J23	188,70	287,63	98,93	238,17
J3	60,00	57,39	-2,61	58,70
J4	111,16	103,58	-7,58	107,37
J5	80,84	123,79	42,95	102,32
J6	322,39	308,06	-14,33	315,22
**Mean**	**119,6**	**173,8**	**54,2**	**146,7**
**DP**	**68,9**	**100,1**	**73,2**	
SEM	16,7	24,3		
Variance	4745	10024	146,4379	200,66
				-92,22

**Mean**
**DP**
SEM
Variance

Lin Coneficient
0,77345013

Results
Dia 1

Statistcs	
R multiplo	0,68207195
R-square	0,465222145
R-sguare aj	0,429570288
Error	52,0284362
N	17

145,3451

ANOVA

	gl	*SQ*	*MQ*	*F*	*F of significance*
Regression	1	35323,17786	35323,18	13,0490298	0,002560033
Residue	15	40604,3726	2706,958		
Total	16	75927,55046			

	Dia 1					
	antecubita	ear lobule	Chance	mean		
J13	120,00	336,92	216,92	228,46		
J17	100,51	143,59	43,08	122,05		
J19	41,31	55,08	13,77	48,20		
J2	133,89	83,37	-50,53	108,63		
J21	119,08	177,71	58,63	148,40		
J22	98,93	263,82	164,89	181,37		
J24	36,92	123,08	86,15	80,00		
J7	111,04	161,19	50,15	136,12		
J10	143,28	250,75	107,46	197,01		
J14	204,18	297,31	93,13	250,75		
J15	75,22	10,75	-64,48	42,99		
J20	85,16	170,32	85,16	127,74		
J23	188,70	287,63	98,93	238,17		
J3	60,00	57,39	-2,61	58,70		
J4	111,16	103,58	-7,58	107,37		
J5	80,84	123,79	42,95	102,32		
J6	322,39	308,06	-14,33	315,22		
	212,31	350,77	138,46	281,54		
	164,50	525,00	360,50	344,75		
	340,33	293,11	-47,21	316,72		
	232,42	333,47	101,05	282,95		
	381,07	608,24	227,18	494,66		
	137,40	252,82	115,42	195,11		
	254,36	508,72	254,36	381,54		
	376,12	616,12	240,00	496,12		
	343,88	616,12	272,24	480,00		
	809,55	1097,31	287,76	953,43		
	411,94	673,43	261,49	542,69		
	166,45	387,10	220,65	276,77		
	390,23	791,45	401,22	590,84		
	224,35	266,09	41,74	245,22		
	492,63	553,26	60,63	522,95		
	323,37	462,32	138,95	392,84		
	368,96	512,24	143,28	440,60		
	180,00	290,77	110,77	235,39		
	164,10	334,36	170,26	249,23		
	171,15	179,02	7,87	175,09		
	174,32	277,89	103,57	226,11		
	133,74	364,58	230,84	249,16		
	120,00	218,02	98,02	169,01		
	106,67	334,36	227,69	220,52		
	146,87	358,21	211,34	252,54		
	150,45	272,84	122,39	211,65		
	214,93	333,13	118,20	274,03		
	558,81	601,79	42,98	580,30		
	207,76	343,88	136,12	275,82		
	141,29	247,74	106,45	194,52		

	120,92	549,62	428,70	335,27	
	88,70	200,87	112,17	144,79	
	275,37	495,16	219,79	385,27	
	204,63	267,79	63,16	236,21	
Mean	**212,20**	**342,59**	**130,39**	**277,39**	**Lin**
	21208,1	42707,49827	111,22		**0,796703601**
			222,440583	352,83	
				-92,05	
		130,39			
		1,614452586			
		161,4452586			

24h			
antecubital	ear lobule	Chance	mean
212,31	350,77	138,46	281,54
164,50	525,00	360,50	344,75
340,33	293,11	-47,21	316,72
232,42	333,47	101,05	282,95
381,07	608,24	227,18	494,66
137,40	252,82	115,42	195,11
254,36	508,72	254,36	381,54
376,12	616,12	240,00	496,12
343,88	616,12	272,24	480,00
809,55	1097,31	287,76	953,43
411,94	673,43	261,49	542,69
166,45	387,10	220,65	276,77
390,23	791,45	401,22	590,84
224,35	266,09	41,74	245,22
492,63	553,26	60,63	522,95
323,37	462,32	138,95	392,84
368,96	512,24	143,28	440,60
**331,2**	**520,4**	**189,3**	**425,8**
**159,1**	**213,6**	**117,3**	
38,6	51,8		
25301	45616	234,5085829	423,79
			-45,23

**Mean**
**DP**
SEM
Variance

Results

Lin Coneficient

0,84832

Statistcs		
R multiplo	0,841390655	
R-square	0,707938235	157,1544102
R-sguare aj	0,68846745	
Error	88,78178131	
N	17	

ANOVA

	gl	*SQ*	*MQ*	*F*	*F of significance*
Regression	1	286589,0751	286589,1	36,359	2,30513E-05
Residue	15	118233,0704	7882,205		
Total	16	404822,1455			

Results

Statistcs	
R multiplo	0,856365
R-square	0,733361
R-sguare ajusted	0,72792
Error	75,9625
N	51

ANOVA

	gl	*SQ*	*MQ*	*F*	*F of significance*
Regression	1	777660	777660,0328	134,7694	1,13249E-15
Residue	49	282744,8	5770,301878		

Total	50	1060405

	Coeficientes	*Erro padrão*	*Stat t*	*valor-P*	*95% inferiores*
intersection	5,458111	20,74355	0,263123301	0,793559	-36,2276108
Variável X 1	0,603473	0,051983	11,60902169	1,13E-15	0,499009014

48h			
antecubital	ear lobule	Chance	mean
180,00	290,77	110,77	235,39
164,10	334,36	170,26	249,23
171,15	179,02	7,87	175,09
174,32	277,89	103,57	226,11
133,74	364,58	230,84	249,16
120,00	218,02	98,02	169,01
106,67	334,36	227,69	220,52
146,87	358,21	211,34	252,54
150,45	272,84	122,39	211,65
214,93	333,13	118,20	274,03
558,81	601,79	42,98	580,30
207,76	343,88	136,12	275,82
141,29	247,74	106,45	194,52
120,92	549,62	428,70	335,27
88,70	200,87	112,17	144,79
275,37	495,16	219,79	385,27
204,63	267,79	63,16	236,21
**185,9**	**333,5**	**147,7**	**259,7**
**106,5**	**117,7**	**96,9**	
25,8	28,5		
11336	13848	193,7018	341,37
			-46,04

Results

Lin Coneficient

0,98024

Statistcs		
R multiplo	0,630687	
R-square	0,397766	179,4478
R-sguare aj	0,357617	
Error	85,33616	
N	17	

ANOVA

	gl	*SQ*	*MQ*	*F*	*of significance*
Regression	1	72147,14	72147,14	9,907245	0,006639
Residue	15	109233,9	7282,261		
Total	16	181381,1			

5% superior	*ferior 95,0%*	*perior 95,0%*
47,14383	-36,2276	47,14383
0,707937	0,499009	0,707937
